# Surgical operation combined with bronchoscopy in the treatment of fungal empyema: 5 cases report

**DOI:** 10.1097/MD.0000000000031080

**Published:** 2022-10-21

**Authors:** Shunxin Xin, Yongyong Wu, Zhongliang He, Xueming He, Lei Wang, Yaoli Qi

**Affiliations:** a Department of Cardiothoracic Surgery, Hangzhou, Zhejiang Province, China; b Department of Cardiothoracic Surgical Nursing, Hangzhou, Zhejiang Province, China; c Tongde Hospital of Zhejiang Province, Hangzhou, Zhejiang Province, China.

**Keywords:** fungal empyema, individualization, multidisciplinary, surgical treatment

## Abstract

Fungal empyema is a chronic refractory disease. It is difficult to control thoracic infection, and it is faced with the problem of recurrence. How to control the infection and reduce the probability of recurrence is a difficult problem. Surgical operation combined with endobronchial therapy was used to control infection, seal the fistula and eliminate residual cavity and achieved good results.

**Patient concerns:**

A total of 5 patients with fungal empyema were treated from 2019 to 2021, aged 27 to 72 years, with an average age of 54.8 ± 7.6 years. Two cases were on the left side and 3 cases on the right side.

**Diagnosis:**

While meeting the diagnostic criteria of empyema, the diagnosis of fungus in pus culture or the discovery of fungus in deep tissue pathology confirmed the diagnosis of fungal empyema in the 5 cases.

**Interventions:**

Through surgical operations combined with bronchoscopy and individualized treatment, the infection was controlled, the fistulas were blocked, and the pus cavity was filled.

**Outcomes:**

After 11 to 30 months of follow-up, the muscle flap in the abscess cavity was mildly atrophied, and there was no recurrence of empyema. Three patients who completed the second-stage operation had their chest tubes removed and returned to normal life. The 2 patients who did not complete the second-stage operation had no recurrence of thoracic infection and no recurrence of cough or fever, and their quality of life was greatly improved.

**Lessons:**

Surgical operation combined with bronchoscopy is a reliable method for the treatment of fungal empyema, which can find and plug the fistula more efficiently and eliminate the residual cavity by surgery to avoid recurrence. Therefore, it is a recommended treatment method.

## 1. Introduction

Empyema is a serious respiratory disease that mostly occurs after lung resection, with an incidence of 1% to 10%.^[1]^ The pathogens are mainly bacteria and are rarely caused by fungi.^[2,3]^ At present, there is no unified treatment method.^[4]^ In addition to intravenous antifungal treatment, common surgical treatment methods include chest tube drainage, pleural cavity irrigation, empyema fibrous plate stripping, empyema debridement and drainage, and chest wall fenestration among others. However, the overall efficacy is still poor. From 2019 to 2021, we treated 5 patients with fungal empyema, applied surgical treatment, and achieved satisfactory results. The clinical experience is summarized as follows.

## 2. Case presentation

### 2.1. Clinical data

A total of 5 patients with fungal empyema were admitted from 2019 to 2021. Among them, there were 4 males and 1 female; aged 27 to 72 years, mean (54.8 + 7.6) years old; 2 cases on the left side and 3 cases on the right side. Causes of empyema: 4 cases after lung cancer surgery and 1 case after lung abscess surgery; all had symptoms of fever, cough, and expectoration, and some patients had symptoms such as chest pain, low back pain, chest tightness, and shortness of breath. The disease duration ranged from 1 month to 1 year, with an average of 6.6 ± 2.0 months; 4 patients underwent 2 operations, and 1 patient underwent 3 operations. The volume of the abscess cavity was measured during the first stage of debridement and drainage. The smallest volume was 50 mL, and the largest was more than 500 mL; all patients had bronchopleural fistulas, and the diameters of the fistulas measured by bronchoscopy ranged from 1 to 3 mm. Fungi were cultured. Two cases were found to have fungi in pus culture and pathology, both of which were *Aspergillus*. One case was pathologically found to have fungi, and the specific bacterial species could not be identified. One case was positive in pus culture and sputum culture for *Candida albicans*. The last case was positive for *Candida parapsilosis* by blood culture and pus culture (Table 1).

**Table 1:** Clinical data of 5 patients with fungal empyema.

Name	Patient A	Patient B	Patient C	Patient D	Patient E
Gender	Female	Male	Male	Male	Male
Age(yrs)	27	72	57	63	55
Inducement	Left lung cancer surgery	Lung abscess surgery	Right middle and lower lung cancer surgery	Upper left lung cancer surgery	Pneumonectomy for left squamous cell lung cancer
Underlying diseases	None	Diabetes, high blood pressure, intestinal obstruction	None	None	None
Course of disease(months)	10	4	More than 12	6	1
Location	Upper left lung	Right lung	Right lower lung	Upper left lung	Left lung
Cavity volume (ml)	90	80	50	120	600
Fistula (mm)	1	1	1	2	3
Pathogen	Aspergillus	*Candida parapsilosis*, *Acinetobacter baumannii*, *Enterococcus faecalis* (Group D), *Klebsiella pneumoniae*	Fungus (specifically unknown)	*Aspergillus*	*Candida albicans*
Chief complaint	Fever, cough	Fever	Fever, cough	Fever, cough	Fever, cough
Surgery	**First stage:** debridement and drainage of empyema and intraoperative bronchoscopy and Bronchopleural fistula closure. **Second stage:** Pectoralis major muscle flap packing	**First stage:** debridement and drainage of empyema. **Second stage:** Latissimus dorsi pedicled muscle flap transplantation and tamponade	**First stage:** debridement and drainage of empyema. **Second stage:** Right free lateral femoral myocutaneous flap transplantation and tamponade	**First stage:** debridement and drainage of empyema; OWT	**First stage:** debridement and drainage of empyema and Bronchopleural fistula closure

OWT = open window thoracostomy.

### 2.2. Staged surgical treatment

Hypoalbuminemia was actively corrected, infection was controlled, anemia was corrected, and intravenous nutrition was actively provided before surgery. Preoperative routine computerized tomography (CT)-guided abscess puncture and catheterization to drain pus can also provide guidance for the intraoperative search for abscesses. Surgery is usually performed in 2 stages.

One-stage debridement and drainage were combined with intraoperative bronchial examination, namely, empyema debridement closed drainage + bronchoscopy. During the operation, part of the ribs near the abscess cavity were excised, the pus coating was fully removed, the pus was cleaned, the abscess cavity was perfused with normal saline for leak testing, combined with intraoperative bronchoscopy to find the fistula, and the size of the fistula was measured under bronchoscopy and in the operative field. Vaseline gauze was used to temporarily seal the fistula to ensure no air leakage, and then the wound and abscess cavity were washed with a large amount of normal saline, povidone-iodine and hydrogen peroxide until the flushing fluid was clear, and the flushing fluid was absorbed. Then, a 50 mL syringe was used to inject physiological saline into the abscess cavity, and the volume of the abscess cavity was measured indirectly. After suctioning the physiological saline, 2 drainage tubes were placed, and the tissue was sutured to the skin layer by layer. In the first-stage operation, if the infection of the abscess cavity is severe, the local pus coating is thick and there is a large amount of necrotic tissue. Therefore, when debridement and closed drainage are expected to fail to control the infection, conversion to open window thoracostomy (OWT) is performed. In addition, if the patient continued to have fever after closed drainage of empyema debridement, there was pus from the chest tube, and the white blood cells continued to rise, the operation was performed again, and OWT was performed.

Under general anesthesia, intraoperative bronchoscopy was performed to find the fistula. If there is no obvious fistula, there are 2 ways to find the potential fistula: Inject the methylene blue solution into the chest cavity: enter the bronchus with a possible fistula through the bronchoscope, and slowly inject the diluted methylene blue solution into the abscess cavity (methylene blue: normal saline = 20 mg: 250 mL). If the methylene blue solution is seen in the bronchus, it is determined that there is a fistula, and this step is repeated until all bronchial fistulas are found. Guide wire exploration: the bronchoscope enters the suspicious bronchus, and a soft-tipped guide wire with a certain hardness enters the line of sight through the working hole of the bronchoscope. The guide wire is used to explore the bronchial stump or the suspicious fistula. If the guide wire can pass easily, it proves that there is a fistula. Through the above 2 methods, most potential fistulas can be found, and the detection rate of the fistulas can be greatly improved. Among the 5 patients in this group, there was 1 left main bronchus stump fistula with a diameter of approximately 3 mm, 1 right middle bronchial stump fistula with a diameter of approximately 1 mm, and 1 left upper lobe superior lingual branch stump fistula with a diameter of 1 mm, all identified by the methylene blue solution leak test method. No fistula was found during bronchoscopy in 1 case; instead, the fistula was found on the surface of the visceral pleura during long-term dressing changes after OWT, with a diameter of approximately 2 mm. In the last case, a fistula was found on the surface of the lung, with a diameter of approximately 1 mm.

In the second stage, bronchoscopy interventional therapy was combined with surgical treatment, namely, bronchoscopy interventional fistula closure with muscle flap transplantation and packing. Closure of the fistula is usually performed at the same time as the second-stage operation. The closure method of the fistula depends on the size of the fistula: for a large fistula (6–20 mm), an atrial septal defect occluder is the first choice, the small head is indwelled in the airway, the large head is indwelled in the abscess cavity; for small fistulas (1–6 mm) one-way valves were placed in a positive direction to seal the fistulas so that the secretions in the airway could not enter the abscess cavity. Granulation tissue hyperplasia near the valves completely closed them and isolated the abscess cavity and the airway.

Depending on the size and location of the abscess cavity and whether the thoracodorsal artery is unobstructed, pedicled muscle flaps (latissimus dorsi, pectoralis major, serratus anterior, etc) or free tissue flaps (anterolateral thigh myocutaneous flap) could be selected to fill the abscess cavity. After the first-stage operation, when the patient has no obvious fever, no pus discharge from the chest tube, the routine blood examination, and CRP are basically normal, and moderate to severe anemia and severe hypoalbuminemia are corrected, the second-stage operation can be performed. Selected muscles for pedicled muscle flap transplantation include the latissimus dorsi, pectoralis major, serratus anterior, rectus abdominis, etc. The required muscles are determined according to the location and volume of the abscess cavity. If the above muscles have been cut or the volume is insufficient, or the pedicled muscle flap was transplanted and packed, but the operation failed, and there was still chest infection, an anterolateral thigh myocutaneous flap was selected to fill the abscess.

## 3. Results

**Figure 1.**
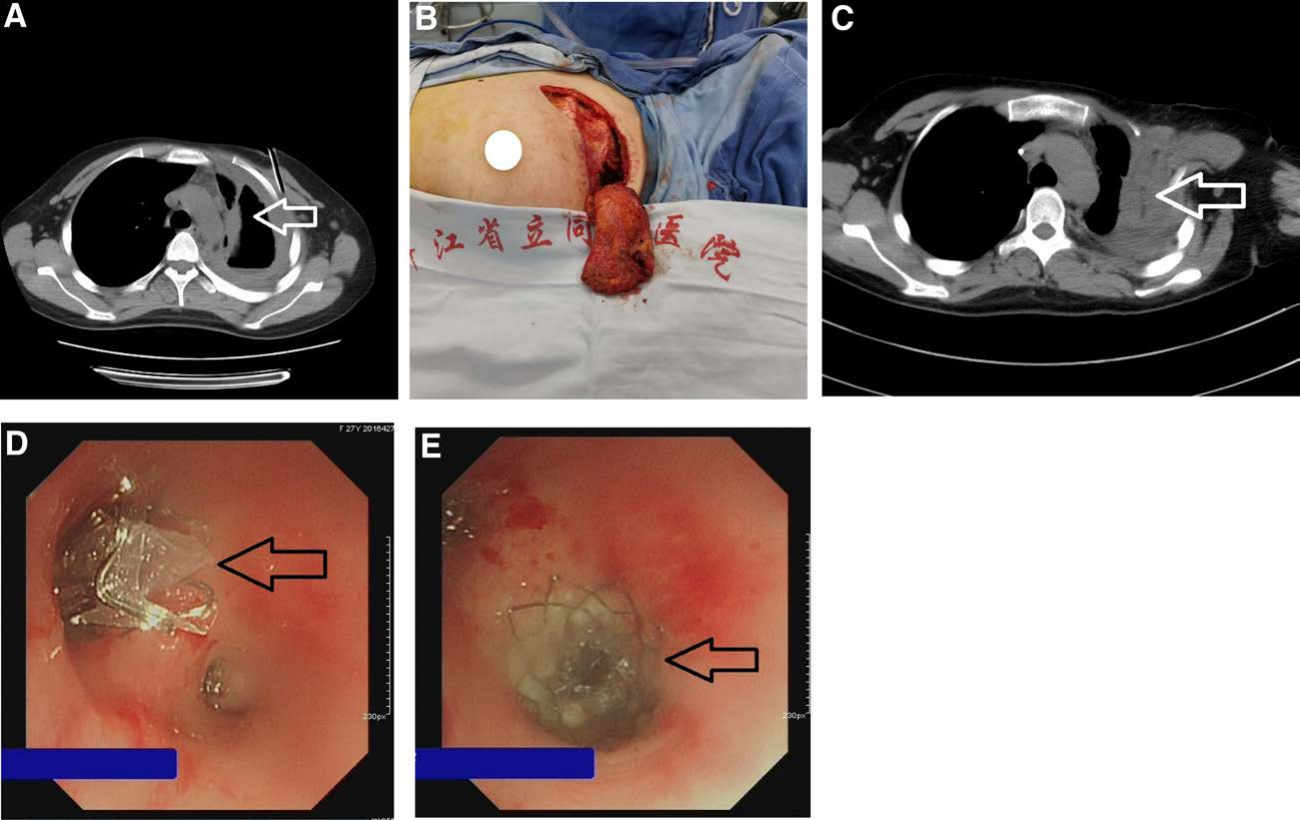
The abscess cavity in the upper field of the left lung of patient A was approximately 90 mL in size (arrow of Figure a, drainage tube can be seen in the anterior chest wall). The abscess cavity was filled with the ipsilateral pectoralis major muscle (Figure b). Chest CT was reexamined 9 days after the operation, and no abscess cavity was found (Figure c). Fistulas of apicoposterius segmentum and anterius segmentum were blocked for a larger bronchial one-way valve (Figure d). Fistula of superior lingular segment was blocked for a smaller one (Figure e).

**Figure 2.**
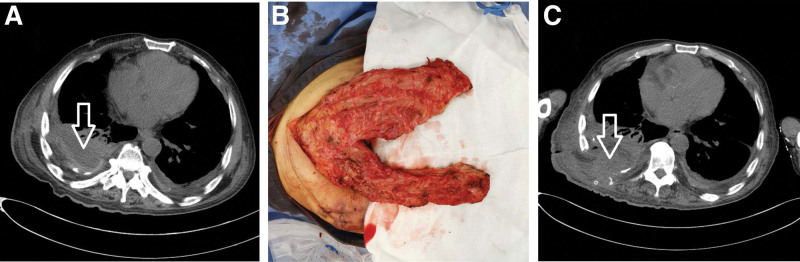
Patient B had an 80 mL abscess cavity on the dorsal side of the right lung (arrow in Figure a), and the ipsilateral latissimus dorsi muscle was selected to fill the abscess cavity (Figure b). Chest CT was reviewed 19 days after the operation and showed that the abscess cavity had completely disappeared (Figure c).

**Figure 3.**
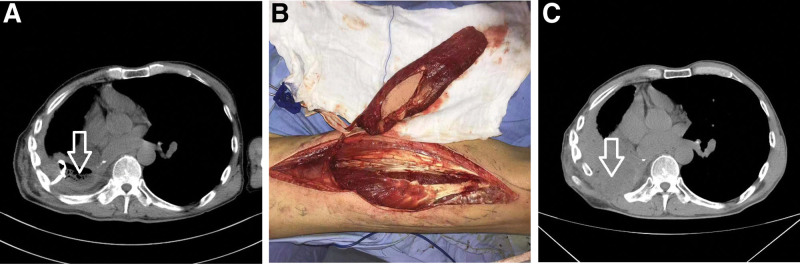
Patient C had empyema caused by right middle and lower lung squamous cell carcinoma after surgery, and the abscess cavity was approximately 50 mL in size (abscess cavity at the arrow in Figure a, with drainage tube visible inside). The right free lateral thigh musculocutaneous flap was selected (Figure b), the abscess cavity was transplanted and filled, and the abscess cavity disappeared completely 15 mo after surgery (Figure c).

Of the 5 patients, 3 patients completed the second-stage operation, and 2 patients only completed the first-stage operation. Among the patients who completed the second-stage operation, the abscess cavity of patient A was approximately 90 mL. The abscess cavity of patient A was about 90 mL, and there were 3 bronchopleural fistulas located in the apicoposterius segmentum, anterius segmentum and superior lingular segment of the left lung respectively. Two 1-way valves were used to plug the fistula (fistulas of apicoposterius segmentum and anterius segmentum were blocked for the larger one). No air leakage was found in the test after the plug. The ipsilateral pedicled pectoralis major muscle flap was used to fill the abscess cavity. The filling effect was good, and the empyema did not recur during postoperative follow-up (Fig. 1). The volume of the abscess cavity of patient B was 80 mL, and the fistula was small (1 mm). The fistula was closed by barbed suture, and the right-side empyema debridement and pedicled muscle flap of the latissimus dorsi was performed, and the effect of filling the abscess cavity was good (Fig. 2). Patient C had a previous thoracotomy and the thoracodorsal artery had been severed, so the latissimus dorsi muscle could not be used. The right free lateral femoral myocutaneous flap was transplanted and tamped, and the fistula was closed by suturing during the operation. During the operation, the dorsal thoracic artery and the descending branch of the lateral femoral artery were anastomosed, and 2 veins were anastomosed. The blood supply of the muscle flap was good, and the abscess cavity completely disappeared. Follow-up over 2 years showed no recurrence (Fig. 3). Among the 2 patients who only completed the first-stage operation, patient E was unable to completely fill the abscess cavity due to the large abscess cavity (600 mL); only the fistula was sealed with a bronchoscope, and the fistula did not leak after the operation. Due to the discharge of pus from the chest tube after debridement and closed drainage, patient D was given salvage thoracic fenestration and the dressing was changed in the abscess cavity every day after surgery, and the second-stage operation was performed at an appropriate time after infection control.

CT = computerized tomography.

CT = computerized tomography.

## 4. Discussion

Empyema is mainly caused by bacteria and is rarely caused directly by fungi (e.g., *Aspergillus*).^[5]^ Fungal empyema accounts for only 2% to 6% of postpneumonectomy empyema cases.^[2,3]^ Although the incidence of fungal empyema is not high, the mortality rate is high.^[2,6]^ Known risk factors associated with fungal empyema (fungal infection of the lungs) include tuberculosis, lung abscesses, lung cysts, bronchiectasis, thoracic intubation or drainage, pneumonectomy, and lung cancer. Systemic factors include diabetes, the use of chemotherapy drugs, and the use of glucocorticoids.^[6–8]^ In recent years, the incidence of pulmonary fungal infection has risen rapidly,^[9]^ primarily due to the application of broad-spectrum antibiotics, intravascular catheters, intravenous hypernutrition, severe cases and the increase in immunosuppressed patients.^[10,11]^ All 5 patients in this group had a history of pulmonary surgery (4 cases of lung cancer and 1 case of lung abscess), and 1 patient had diabetes, and all had high risk factors for fungal infection.

There are no uniform diagnostic criteria for fungal empyema. Ko et al^[2]^ and Lee et al^[12]^ required that while meeting the diagnostic criteria of empyema, the diagnosis of fungus in pus culture or the discovery of fungus in deep tissue pathology can confirm the diagnosis, while fungi in sputum culture alone cannot represent fungal infection in the abscess cavity.^[2]^ In this group of 5 patients, fungal infection was confirmed by pus culture and postoperative pathology, and the diagnosis was clear.

As a disease with a high mortality rate, there is currently no standard treatment for fungal empyema. Voriconazole has been recommended for the first-line treatment of invasive aspergillosis.^[13]^ For patients with a poor response to voriconazole, amphotericin B, echinocandins, or a combination of both can be used.^[13]^ For patients with fungal empyema, systemic application of voriconazole, and micafungin can achieve good drug concentrations in the thoracic cavity.^[14]^ Therefore, voriconazole was selected as the first-line drug for this group of patients, which effectively reduced the fungal load and was well tolerated without adverse reactions.

Even with medical treatment, there are still some patients whose abscess infection cannot be effectively controlled and requires surgical intervention, such as chest tube drainage, empyema debridement and drainage, and OWT.^[7,15–18]^ Prompt surgical treatment is very important for curing fungal empyema, which can effectively reduce the fungal load and improve the success rate of treatment.^[18,19]^ There is no unified consensus on the timing of surgical intervention for fungal empyema. Our experience is that 1 to 2 months after regular antifungal therapy and chest tube drainage, if the chest tube still flows out of pus, the patient still has repeated fever and has an abnormal routine blood examination and CRP, surgical treatment should be performed.

There is no consensus on the surgical selection of fungal empyema.^[12]^ Surgical options include closed chest drainage, pneumonectomy plus thoracoplasty,^[20]^ OWT, omentum tamponade, chest wall muscle tamponade, etc.^[21]^ Closed thoracic drainage is usually used as the initial treatment, which results in minimal surgical injury and can control some patients’ chest infections. Thoracoplasty is now rarely used because of its high level of trauma, severe physical deformities and psychological disorders. Open window thoracostomy (OWT) is widely used, and its safety and effectiveness have been affirmed by most thoracic surgeons.^[21,22]^ It is one of the main surgical methods to control thoracic infection. Omentum and chest wall muscle packing, especially chest wall muscle packing, is increasingly accepted by thoracic surgeons, especially in patients with bronchial stump fistulas.^[21,22]^ Among the 5 patients, 3 patients completed the second-stage operation, the postoperative abscess filling effect was good, there was no recurrence during the follow-up period, and the chest tube was completely removed, which greatly improved the quality of life. The infection of the other 2 patients who had only one-stage operation was also effectively controlled, and the fistula disappeared in 1 patient, which met the patient’s preoperative treatment expectations. Therefore, for patients with fungal empyema, surgical treatment combined with bronchoscopy plays an important role in finding fistula, controlling chest infection and reducing the recurrence of empyema, which is a recommended treatment.

## Acknowledgments

We thank the patients and family members for their participation.

## Author contributions

**Conceptualization:** Shunxin Xin.

**Formal analysis:** Xueming He.

**Methodology:** Yongyong Wu, Zhongliang He.

**Visualization:** Xueming He.

**Writing – original draft:** Shunxin Xin.

**Writing – review & editing:** Lei Wang, Yaoli Qi.
